# Motorcycle Related Injuries among Rural Dwellers in Irrua, Nigeria: Characteristics and Correlates

**DOI:** 10.1155/2013/569103

**Published:** 2013-10-20

**Authors:** A. E. Dongo, E. B. Kesieme, A. Eighemherio, O. Nwokike, E. Esezobor, E. Alufohai

**Affiliations:** ^1^Department of Surgery, Irrua Specialist Teaching Hospital, PMB 08, Irrua, Edo State, Nigeria; ^2^Department of Orthopaedics, Irrua Specialist Teaching Hospital, PMB 08, Irrua, Edo State, Nigeria

## Abstract

*Background*. The escalating use of motorcycle for commercial transportation of commuters and goods has resulted in an increase in morbidity and mortality from road traffic injuries. *Objectives*. To study the characteristics of motorcycle injuries seen in Irrua, Nigeria. *Materials and Methods*. This is a one-year prospective study of all patients seen from January 1, 2009, to December 31, 2009. A structured proforma was filled for all consecutive crash victims involving a motorcycle. *Results*. Motorcycle injuries accounted for 11.6% of attendance in surgical emergency room (142 out of 1,214); 76.8% were males. Amongst victims 47.1% were riders, 42.9%, passengers, and 7.8% pedestrians. Extremity injury accounted for 42.2% while head injury occurred in 21.8%. There were 9 deaths (6.3%). In this study no victim used crash helmet. *Conclusion*. Banning of motorcycle for commercial use and the introduction of tricycles into rural/suburban comminutes may be an important preventive strategy.

## 1. Introduction

Road traffic injuries are a worldwide disaster. The World Health Organisation (WHO) estimates that about 1.2 million die and 50 million are injured yearly [1]. Unfortunately, a disproportionate burden of this injury is currently and will continue to be borne by low income and middle income countries [1, 2]. Some of this increase has been fueled largely by the escalating use of motorcycles for commercial transportation of commuters, goods, and services [3, 4]. Motorcycles are the most dangerous type of motor vehicles to drive accounting for higher rates of crashes and fatalities compared to passenger cars per miles driven [5].

Many reasons have been adduced for the explosion in numbers of Motorcycles on Nigerian roads. These range from the relative low costs of newer Asian models (<$500) to their ability to meander through traffic jams and bad roads [3]. However in rural communities there are no traffic jams. In truth, there is very poor or nonexistent public transportation system to serve rural and suburban communities. The commercial motorcyclist therefore fills a void.

“Okadas” or “One Chance” as commercial motorcycles are eponymously called in Nigeria [6, 7] now serves for inter- and intravillage commuters as well as for conveyance of goods and services. Rural dwellers embrace this service for want of a choice. It is better than use of bicycles or trekking to farms and markets.

The riders exploiting this void do so because of the increasing recognition of the economic empowerment it confers. Within twenty-four hours of commencement of commercial motorcycling, riders often earn enough to feed themselves and a little extra to provide for families. Many local entrepreneurs will provide capital for new motorcycles on hired purchase or simply loan out motorcycles to riders for daily submission of fixed amounts. Either way there is pressure on riders to meet an agreed target and then something extra for the rider. Viewed against the high unemployment rates in our rural communities, this arrangement is magnetic for young men.

This new lure is pulling several young men from farms apprenticeships, and even schools. This largely unregulated use of motorcycles for commercial purposes in Nigeria has many present and future consequences. Many of these riders are not trained [8] and represent a danger to themselves and other road users. Motorcycle injuries now represent one of the commonest causes of hospital admission with attendant loss of life and limbs. 

## 2. Patients and Methods 

This is a prospective study of all patients presenting to the accident and emergency room of Irrua Specialist Teaching Hospital, Irrua, Edo State, Nigeria, after motorcycle injuries between January 1, 2009, and December 31, 2009. Approval was sought and received from the Ethics and Research Committee of Irrua Specialist Teaching Hospital before commencement of the study. Informed consent was obtained in all cases from the patients or their relatives. 

Irrua Specialist Teaching Hospital is a 375-bedded hospital situated in Irrua, a rural community in Midwestern Nigeria. It is about 100 kilometres northwest of the capital city of Benin. It serves principally the Edo central and northern senatorial zones and the neighbouring states of Ondo, Kogi, and Delta. This population is about 3-4 million.

A structured proforma was filled by two of the authors (A. E. Dongo and A. Eighemherio) once a patient with a motorcycle injury was admitted and completed after discharge referral or death. Data collected included demographics, cause of crash, interval before presentation, helmet use, body systems injured, and outcome. Data analysis was done with SPSS 16.

A motorcycle injury is defined as one in which the victim was either riding a motorcycle when the crash occurred or was a pedestrian knocked down by a motorcycle.

## 3. Results 

In the period under review, 1,214 patients presented to the surgical emergency department, 142 (11.6%) were victims of motorcycle injury. There were 109 males (76.8%) and 33 females (33%). Amongst the victims, 67 (47.1%) were riders, 61 (42.9%) were passengers, and 14 (9.8%) were pedestrians. Seven (50%) of pedestrians were less than 20 years (ages 2–13 years) (Table 1). 

The age range was from 2 to 78 years. The mean age (SD) was 33.2 (14.7 years). The peak incidence was 21–30 years (49 patients 34.5%). 

Majority of the victims (67, 47.2%) arrived at the emergency room after 1 hour but before 6 hours after the crash. 58 (40.8%) arrived within the golden hour. Seven patients (4.9%) arrived after 6 hours but within 24 hours. Two patients (1.4%) arrived after 24 hours. The time of arrival of patients (5.6%) was not specified. 

**Figure 1:**
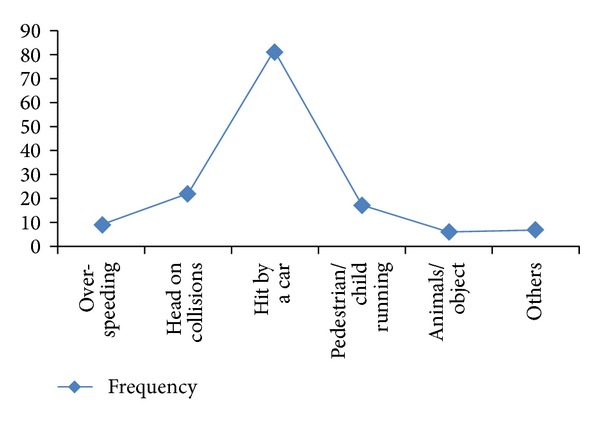
Causes of motorcycle crash.

The commonest cause of crash was identified as being hit by a car in 81 patients (57.0%). The next most frequent cause was collision with another motorcycle (22, 15.3%) followed by pedestrians running into a motorcycle (17, 12.0%), over-speeding/loss of control (9, 6.3%), animal/object on the road (6, 4.2%), and others (7, 4.9%) (Figure 1). 

The commonest and anatomic region injured was musculoskeletal; 60 (42.2%) had fractures of the extremities. 46 (32.4%) had lower limb fractures, 14 (9.8%) had upper limb fractures, 3 (2.1%) had both, and 31 (21.8%) had head/or spinal cord injures. 71 (50%) had soft tissue injuries in various combination with the aforementioned major injuries. Seven (4.9%) had blunt chest trauma. One (0.7%) had blunt abdominal injury with splenic rupture. A further review of 31 head injured victims revealed that riders constituted 21 (67.7%) while passengers were 8 (25.8%). Of the 57 patients with extremity fractures 23 (40.3%) were riders and 27 (47.3%) were passengers. 

The educational level of victims revealed that of the 67 riders, 33 (49.2%) had primary level of education or none at all. 46 (75%) of passengers had at least secondary education with 21 (34.4%) having tertiary education. The occupational status revealed that 42 (29.5%) of the victims were commercial motorcyclists, 31 (21.8%) were students, and 13 (9.1%) were farmers (Table 2).

A review of outcome revealed that 120 (84.5%) were discharged within 2 weeks, while 18 (15.5%) stayed beyond 2 weeks in hospital. A further breakdown showed that while 83 (58.5%) were discharged home, 37 (26.1%) requested for discharge against medical advice and 13 (9.2%) were referred. There were 9 deaths (6.3%) in this series. No victim had any crash helmet on at the time of crash. 

## 4. Discussion 

This study identifies motorcycle crash injury as an important cause of admission into surgical emergency rooms. It contributed 11% of all admissions in the period under review. This provides the first data from our suburban centre and against which future trends may be compared.

There is a male preponderance in this study. This is in agreement with several other reports [3, 4, 9]. It is observed that nearly all commercial motorcyclists are males and riders constituted the single largest risk group (47.7%). This agrees with other studies which identify riders as the majority of motorcycle crash victims presenting to hospitals [3, 10, 11]. The 9.8% of injuries involving pedestrians is one of the lowest observed. It is lower than the 14–22.5% by other workers [3, 10]. About half of the pedestrian victims were children. It has previously been reported that such children victims are often on errands [12] and unsupervised by the road side. The absence of segregated road networks for pedestrians increases the risk of accident.

The commonest cause of motorcycle crash was collision with a car (81, 57%). Vehicles have been reported to contribute majority of motorcycle accidents mainly due to their inability to detect or recognize them in traffic [3]. Contrary to the widespread belief amongst motorcyclists deliberate hostile action by a motorist against a rider is a rare cause of accidents [3]. In the course of this study, we observed many fractious disagreements in the emergency rooms between vehicle owners who were often not hurt and motorcyclists who bear the brunt of these collisions. The motorists often accuse the motorcyclists of always being in a hurry and not paying attention to road signs and other road users, whereas motorcyclists see car owners as not according to them respect as fellow road users. It often reflects a sort of class conflict between the “poor,” working class (motorcyclist) and the “rich” (car owner). Following any road crashes these days, all commercial motorcyclists plying that route often disembark in solidarity with the motorcycle crash victim and harass the car owner involved. Nevertheless, in about 43%, there is no involvement of a car in crashes. These crashes involved motorcyclists alone, either with fellow riders or pedestrians or animals or objects on the way. 

Despite widespread evidence in support of the protective effect of helmet use in motorcycle riders [13] and laws enacted to punish offenders [3], no single victim from this study had a helmet on. Many studies have previously documented low helmet use amongst motorcyclists in Nigeria [11, 14]. Not surprisingly, head injury constituted the 2nd most common anatomic region involved. A Greek study identifies several reasons for low helmet use amongst motorcycle riders like peer pressure, lack of appropriate information, high cost and lack of convenience, disturbance of hearing and vision, or messing up of hair [15]. Attention has also been drawn to underlying sociocultural factors for accident proneness and riding behavior in motorcyclists [16]. Amongst Nigerians, we speculate that different dynamics of a socio-cultural theme may be at play. There is certain tendency towards some superstitious beliefs like risk of mental illness, hypnosis, or disappearance when wearing a bewitched helmet. One state in Nigeria has now requested passengers to carry along their own helmets. It remains to be seen how effective this would be as a public health strategy. However, this may not address the issue of our hot humid environment often cited as discouraging helmet use. While it would be helpful to investigate the sociocultural reasons for noncompliance with motorcycle helmet use in Nigeria, we advocate enforcement of laws concerning helmet use. This is known to improve compliance and results in large public health benefits [17–19].

This study identifies lower limb fracture as the most common severe injury sustained by victims of motorcycle crashes. Just under 1/3 of victims (32.4%) suffered lower limb fractures. About one out of every five victims had head/spinal injuries. In this study, whereas passengers were more prone to extremity fractures, riders have a higher likelihood of head injuries (21/31); a larger study would be required to validate this observation.

Commercial motorcyclists were the single largest occupational risk factor. Just under 30% of victims identify this as their occupation. As 50% of riders had only basic primary education or none at all, it may be inferred that lack of formal education could be a contributory factor to motorcycle crashes. Students were the second commonest occupation of victims. Irrua is near the university town of Ekpoma and motorcycle is a common means of transportation to and from schools. An effective public transportation system for schools will prevent most of these injuries. Farmers represented 13 (9%) of the victims. There were one death and 5 limb fractures amongst them. Three farmers were referred to University of Benin Teaching Hospital for moderate to severe head injuries. Their injuries were often sustained on the way from farm after a hard-day work. Farming is an important means of livelihood in rural Africa [20]. Many such farmers practice subsistence agriculture contributing about 80% of food production in Nigeria [21]. Efforts to stem motorcycle crashes in rural areas may be important in reducing threats to food security.

31 patients (26.1%) requested for discharge against medical advice. This was typically from patients with fractures who preferred traditional bone setters (TBS) to orthodox management of fractures. Although the hazards of TBS have been well documented in the past [22, 23], this practice still holds a major attraction for our patients in the rural areas. Easy accessibility and low charges have been cited as reasons why this practice remains popular amongst our patients with extremity fractures [24]. 

There were 9 (6.3%) mortalities in the series although one major limitation is the absence of a neurosurgeon in this hospital during the period under study. Therefore all patients with moderate to severe head injuries (13, 9.2%) were referred. There was no follow up to determine those who survived. It is likely that a higher mortality could have been observed. 

This study has limitations. It was impossible to corroborate independently the information provided by victims. There is gross underreporting of road crashes by both the police and traffic enforcement agencies as victims frequently leave the scene without giving details to authorities.

This study highlights the dangers inherent in the unbridled use of motorcycles for public transportation in Nigeria. The introduction of tricycles and an effective public transportation system in rural areas will help in preventing most of these injuries. 

## Figures and Tables

**Table 1:** Cross tabulation of status by age group.

	Rider	Passenger	Pedestrian	Total
Age				
<20 yrs	3	9	7	19
20–39	42	27	4	73
40–59	16	21	2	39
>60	4	2	1	7
Not known	2	2	0	4

Total	67	61	14	142

**Table 2:** Cross table of occupation and outcome.

Occupation	Outcome				
	Discharge	Referred	DAMA	Death	Total
Bike riders	25	1	13	3	42
Student	19	3	6	3	31
Trading	12	0	6	0	18
Civil servant	9	2	3	0	14
Farming	4	3	5	1	13
Others	14	4	4	2	24

Total	83	13	37	9	142

DAMA: discharged against medical advice.
